# Immediate Remote Common Femoral Artery Rupture After Vascular Closure Device Deployment

**DOI:** 10.1002/ccr3.72627

**Published:** 2026-05-01

**Authors:** Junghyun Park, Jeffrey Steinberg

**Affiliations:** ^1^ Department of Neurosurgery Kosin University Gospel Hospital Busan Republic of Korea; ^2^ Department of Neurosurgery University of California San Diego (UC San Diego) La Jolla California USA

**Keywords:** angiography, arterial rupture, femoral artery, postoperative complications, retroperitoneal hemorrhage, vascular closure devices

## Abstract

Vascular closure devices may cause life‐threatening femoral artery rupture remote from the puncture site. Sudden hemodynamic instability after device deployment should prompt immediate evaluation for proximal arterial injury, even when femoral access appears technically successful.

## 1 Introduction

Vascular closure devices (VCD) are widely used to achieve hemostasis after angiographic procedures, providing shorter hemostasis time and earlier ambulation compared with manual compression [1]. Although generally safe, VCD‐related complications occur in 2%–5% of cases [2, 3], including pseudoaneurysm formation, arterial occlusion, infection, and rarely, retroperitoneal hemorrhage [2, 3, 4, 5]. Arterial injuries typically occur at or near the puncture tract, and rupture of the proximal femoral artery remote from the access site is exceedingly uncommon.

Most reported cases have involved direct vessel wall disruption at the puncture site [6]. In contrast, rupture of the proximal common femoral artery represents an atypical and potentially unrecognized access‐site complication. Prior literature has focused primarily on retroperitoneal hemorrhage from access‐site trauma, which carries substantial morbidity and mortality [7, 8]. Such bleeding is particularly concerning in neuroendovascular procedures, as abrupt hemodynamic deterioration may critically impair cerebral perfusion and result in secondary neurological injury.

Here, we report a rare case of immediate proximal common femoral artery rupture occurring within minutes of VCD deployment after coil embolization for an unruptured intracranial aneurysm, highlighting the importance of early recognition and prompt endovascular management of this life‐threatening complication.

## 2 Case History and Examination

**FIGURE 1:**
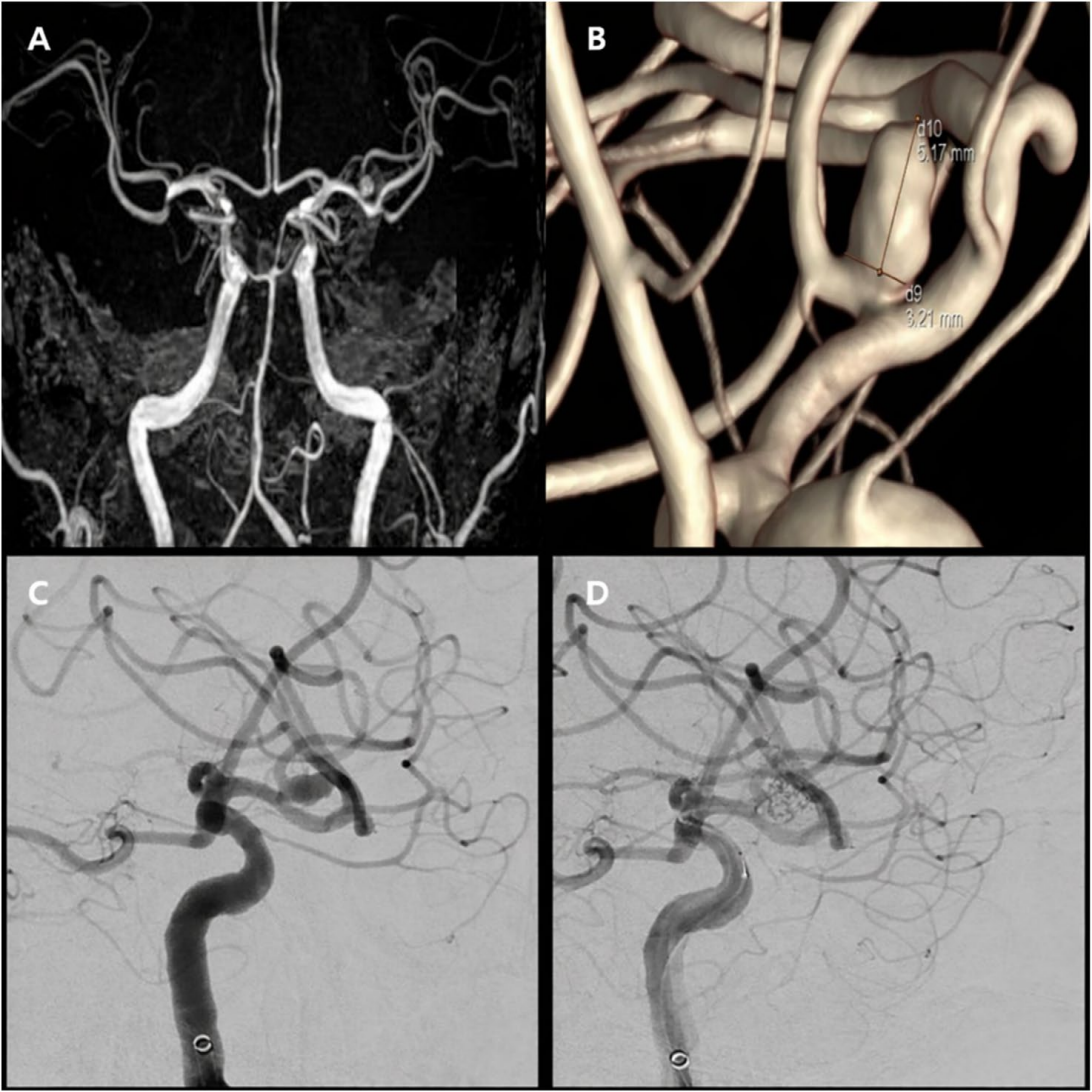
Pre‐ and posttreatment angiographic evaluation of a left middle cerebral artery aneurysm. (A) Time‐of‐flight magnetic resonance angiography demonstrating an unruptured aneurysm of the left middle cerebral artery. (B) Three‐dimensional rotational angiography showing detailed aneurysm morphology (maximum dimensions, 5.2 × 3.2 mm). (C) Working projection digital subtraction angiography used for coil embolization. (D) Posttreatment digital subtraction angiography confirming complete aneurysm occlusion.

A 67‐year‐old man with hypertension, diabetes mellitus, and a history of gastric cancer presented with an unruptured left middle cerebral artery (M1) aneurysm detected on magnetic resonance angiography. Digital subtraction angiography confirmed the lesion, and coil embolization was performed after 2 weeks of dual antiplatelet therapy. MR angiography and 3D rotational angiography demonstrated the aneurysm morphology (Figure 1A,B). Under general anesthesia, right common femoral access was obtained under fluoroscopic guidance, and the aneurysm was successfully treated using a double microcatheter coil embolization technique, achieving complete occlusion (Figure 1C,D). Hemostasis was attempted with a Femoseal VCD, with no immediate technical difficulty noted during deployment.

## 3 Differential Diagnosis, Investigations, and Treatment

**FIGURE 2:**
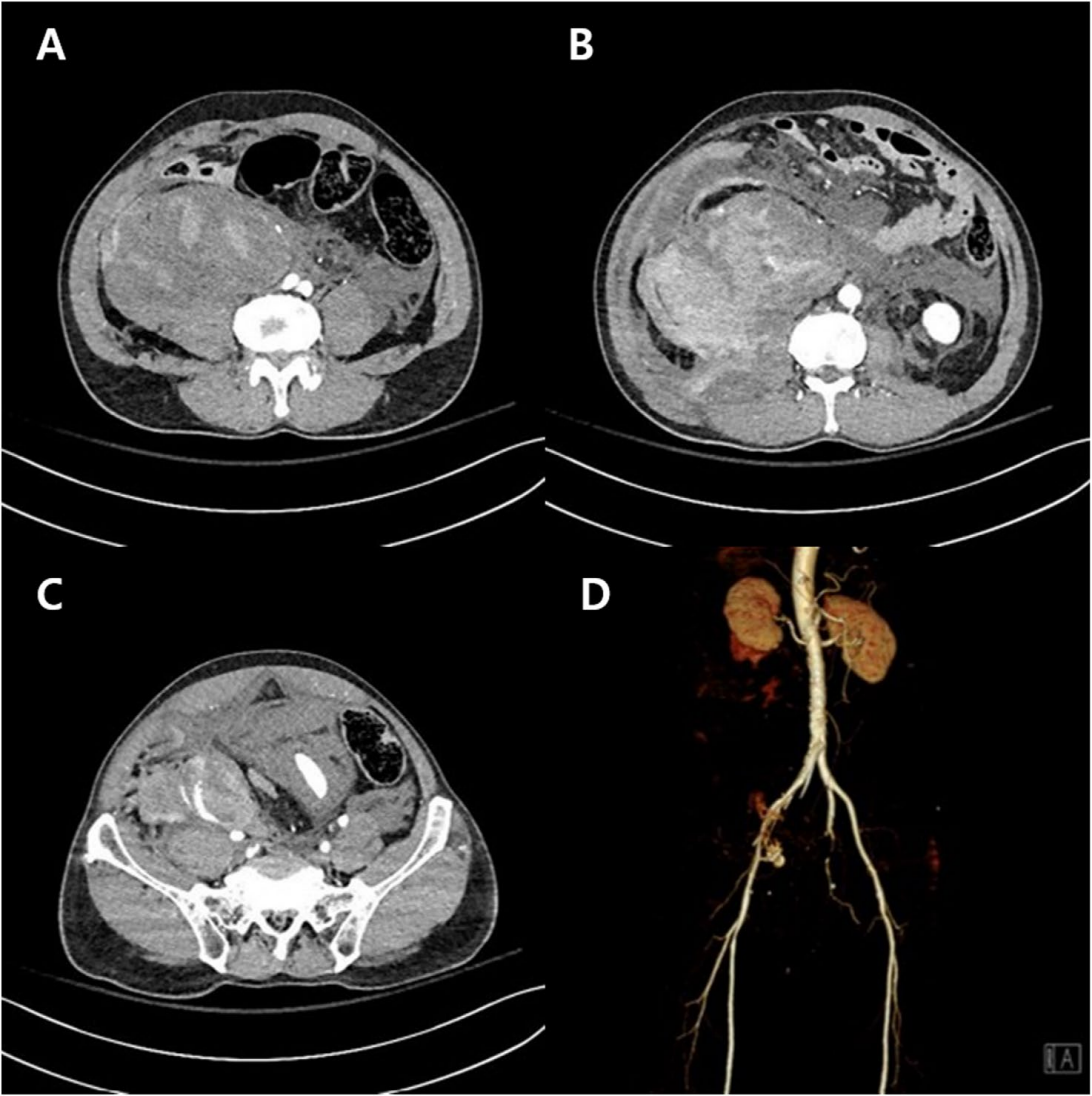
Computed tomography and CT angiography demonstrating retroperitoneal hemorrhage due to proximal common femoral artery rupture. (A–C) Axial computed tomography images showing a massive retroperitoneal hematoma extending along the iliopsoas muscle. (D) Three‐dimensional CT angiography demonstrating active contrast extravasation from the proximal common femoral artery, remote from the femoral puncture tract.

**FIGURE 3:**
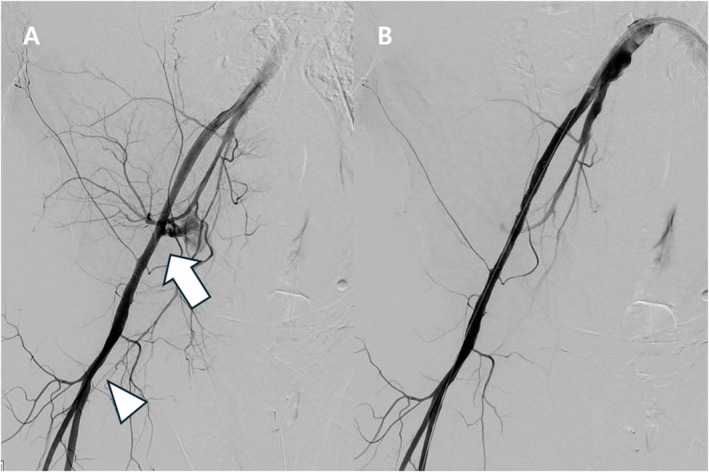
Angiographic demonstration and endovascular repair of proximal common femoral artery rupture. (A) Femoral angiography demonstrating active contrast extravasation from the proximal common femoral artery (arrow). The arrowhead indicates the femoral puncture site used for the neuroendovascular procedure. (B) Post–stent‐graft angiography following deployment of an 8 × 60 mm covered stent‐graft (Fluency Plus; Bard Peripheral Vascular, Tempe, AZ, USA), confirming complete sealing of the rupture with preservation of distal arterial flow.

Within 10 min of arriving on the ward, the patient developed sudden abdominal pain, hypotension, and tachycardia. CT revealed a massive retroperitoneal hematoma (Figure 2A–C), and 3D CT angiography demonstrated active extravasation from the proximal common femoral artery, remote from the puncture tract (Figure 2D). Emergency angiography via the contralateral femoral artery confirmed focal rupture of the proximal common femoral artery (Figure 3A). An 8 × 60 mm Fluency Plus covered stent‐graft was immediately deployed, achieving prompt hemostasis with preservation of distal flow (Figure 3B).

## 4 Conclusion and Results (Outcome and Follow‐Up)

**FIGURE 4:**
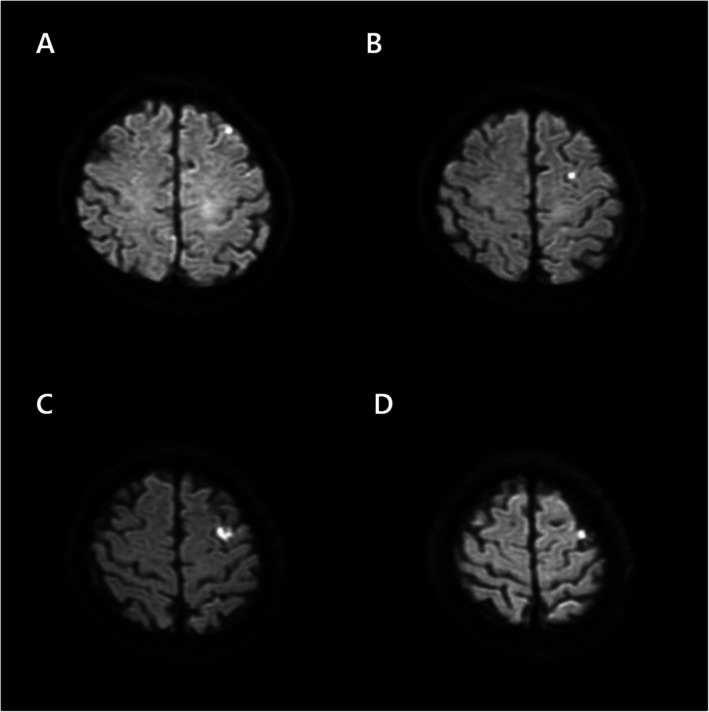
Diffusion‐weighted magnetic resonance imaging demonstrating acute cerebral infarction. (A–D) Diffusion‐weighted images showing high‐signal‐intensity lesions in the cortical–subcortical watershed zone between the anterior cerebral artery and middle cerebral artery territories, involving the superior frontal and parietal lobules.

The patient subsequently developed hemorrhagic shock, acute kidney injury requiring continuous renal replacement therapy, pulmonary edema with bilateral pleural effusions requiring drainage, and dysarthria. Diffusion‐weighted MRI demonstrated an acute watershed infarction (Figure 4). Surgical evacuation of the hematoma was deferred because of hemodynamic instability, and percutaneous drainage was performed after liquefaction on day 7. The patient gradually improved and was discharged after 1 month with only mild residual thigh sensory deficit.

## 5 Discussion

Immediate proximal femoral artery rupture remote from the puncture tract after VCD deployment is exceedingly rare. While VCD‐related complications occur in approximately 2%–5% of cases [2, 3, 4, 5], most reported arterial injuries involve the puncture site itself, and acute rupture at a proximal site remote from access has rarely been described. Prior reports have documented delayed retroperitoneal hematoma [9], femoral artery occlusion after suture‐mediated closure [10], and acute occlusion after Angio‐Seal use [11], but none describe immediate proximal rupture with rapid hemodynamic collapse as observed in this case.

**FIGURE 5:**
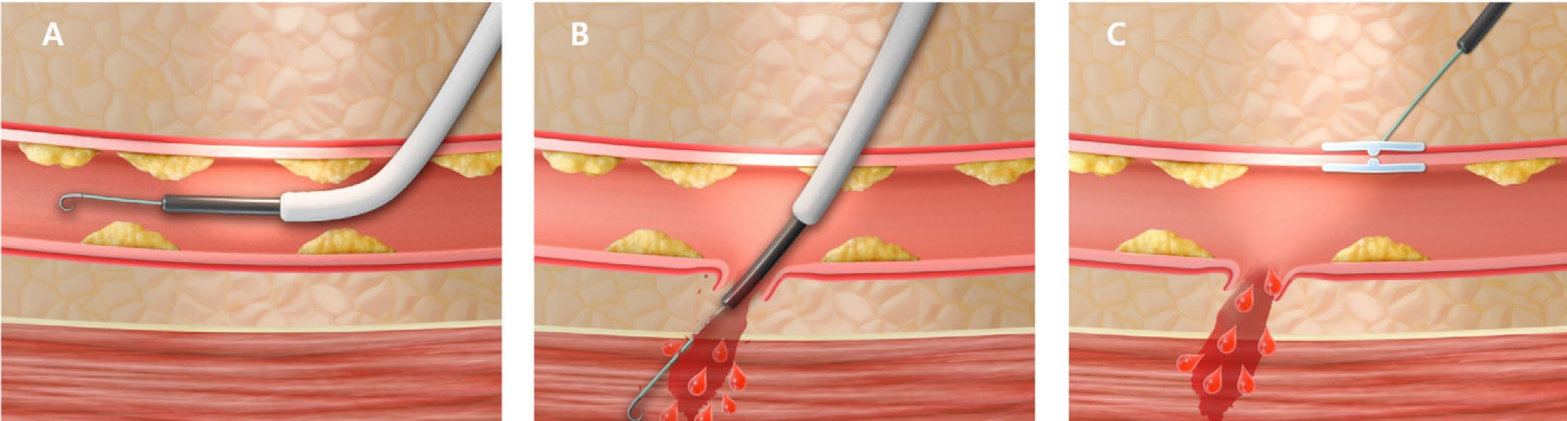
Proposed mechanism of proximal common femoral artery rupture associated with vascular closure device deployment. (A) Proper intraluminal positioning of the anchor within the true lumen of the common femoral artery. (B) A subtle oblique entry angle may cause the anchor to strike the contralateral arterial wall, producing a deep mural injury that rapidly progresses to full‐thickness rupture under arterial pressure. (C) Massive extraluminal hemorrhage occurs from the ruptured contralateral wall, while the original arteriotomy is sealed by the collagen plug, allowing hemorrhage to progress unnoticed. This schematic illustration is a conceptual representation created for explanatory purposes.

A plausible explanation is a two‐hit mechanism. First, underlying arterial fragility associated with advanced age, hypertension, diabetes, and subclinical atherosclerosis may predispose the vessel wall to injury [12, 13, 14]. Second, mechanical stress generated during anchor deployment or axial traction of the VCD—particularly if positioned slightly cranial to the arteriotomy or against a calcified arterial segment—may result in focal intimal injury that propagates under arterial pressure, leading to full‐thickness rupture. The close temporal relationship between device deployment and clinical collapse strongly implicates the VCD as the precipitating factor. The proposed mechanism is illustrated in Figure 5. Ultrasound‐guided femoral artery access is increasingly recommended as a standard approach in contemporary vascular and endovascular practice, as it enables real‐time visualization of arterial anatomy, including the femoral bifurcation and the presence of calcified plaques, thereby reducing access‐site complications [15, 16, 17]. Furthermore, ultrasound guidance during vascular closure device deployment may help ensure accurate positioning of the device and allow immediate assessment of arterial wall integrity, potentially reducing device‐related vascular injury [18]. Recent evidence specifically supports ultrasound‐guided deployment of Femoseal, demonstrating improved accuracy and reduced complications [19].

In the present case, the procedure was performed under fluoroscopic guidance without ultrasound, which represents an important limitation of this case, as real‐time imaging might have reduced the risk of unintended arterial wall injury and improved the accuracy of both arterial access and device deployment [15, 16, 17, 18, 19].

In this context, ultrasound guidance might have helped identify suboptimal arterial segments, such as calcified or noncompressible regions, potentially preventing this sequence of injury.

In addition, careful pre‐procedural assessment of the femoral access site is essential. In patients with significant comorbidities or suspected vascular disease, evaluation of arterial anatomy—potentially including ultrasound or CT angiography—and in selected cases, consultation with a vascular specialist may help determine the safest access strategy and reduce the risk of access‐related complications.

Notably, the rupture occurred at a site anatomically distant from the arteriotomy, suggesting that the mechanism of injury may involve intraluminal device–arterial wall interaction rather than direct puncture‐related trauma.

Early recognition of this complication is critical. The combination of abdominal pain, hypotension, and tachycardia after femoral closure should immediately raise suspicion for retroperitoneal hemorrhage [20, 21]. CT angiography establishes the diagnosis, while catheter angiography enables both localization and definitive treatment [22]. Covered stent‐grafts are increasingly used for iatrogenic femoral rupture, particularly in unstable or high‐risk patients, and prior reports demonstrate high rescue success in superficial or deep femoral injuries [23, 24, 25, 26]. In neuroendovascular patients, prompt restoration of hemodynamic stability is especially important to prevent secondary cerebral ischemic injury, as illustrated by the watershed infarction observed in this case. This case underscores that access‐site complications, even when remote from the puncture tract, can have direct neurological consequences if not recognized and treated immediately.

## Author Contributions

**Junghyun Park:** conceptualization, data curation, investigation, visualization, writing – original draft. **Jeffrey Steinberg:** supervision, validation, writing – review and editing.

## Funding

The authors have nothing to report.

## Ethics Statement

Ethical approval was not required for this case report in accordance with institutional guidelines.

## Consent

Written informed consent was obtained from the patient for publication of this case report and the accompanying images.

## Conflicts of Interest

The authors declare no conflicts of interest.

## Data Availability

The authors have nothing to report.
